# Magnetic *ε*-Phosphorene for Sensing Greenhouse Gas Molecules

**DOI:** 10.3390/molecules28145402

**Published:** 2023-07-14

**Authors:** Zengyao Wang, Hao Wu, Qingyun Wu, Yi-Ming Zhao, Lei Shen

**Affiliations:** 1 Engineering Science Programme, Faculty of Engineering, National University of Singapore, Singapore 117575, Singapore; 2 Department of Mechanical Engineering, National University of Singapore, Singapore 117575, Singapore; 3 Science, Mathematics and Technology, Singapore University of Technology and Design, 8 Somapah Road, Singapore 487372, Singapore

**Keywords:** gas sensor, toxic gases, phosphorene, 2D materials, DFT calculations

## Abstract

It is critical for gas sensors that sense greenhouse gas molecules to have both good sensitivity and selectivity for water molecules in the ambient environment. Here, we study the charge transfer, IV curves, and electric field tuning of vanadium-doped monolayer ϵ-phosphorene as a sensor for NO, NO_2_, and H_2_O gas molecules via first-principle and transport calculations. We find that the paramagnetic toxic molecules of NO and NO_2_ have a high adsorption energy on V-ϵ-phosphorene, which originates from a large amount of charge transfer driven by the hybridisation of the localised spin states of the host with the molecular frontier orbital. Using the non-equilibrium Green’s function, we investigate the IV responses with respect to the adsorption of different molecules to study the performance of gas molecule sensors. Our IV curves show a larger amount of changes in resistance of the paramagnetic NO and NO_2_ than nonmagnetic H_2_O gas molecules, suggesting both sensitivity and selectivity. Moreover, our calculations show that an applied external electric field (gate voltage) can effectively tune the amount of charge transfer. More charge transfer makes the sensor more sensitive to the molecule, while less charge transfer can reduce the adsorption energy and remove the adsorbed molecules, allowing for the repeated use of the sensor.

## 1. Introduction

In the pivotal year of 2022, the United Nations Environment Programme (UNEP) orchestrated a significant event, the Stockholm+50 conference [1], emphasising the reduction of greenhouse and toxic gases in the forthcoming decade. According to alarming statistics, toxic gases such as nitrogen oxides can lead to a variety of serious diseases. Monitoring these hazardous gases is essential to safeguard human life and achieve the Sustainable Development Goals. Innovative gas sensors employ two primary mechanisms to monitor toxic gases: surface-absorbed oxygen ions and charge transfer mechanisms [2,3,4]. These cutting-edge devices convert interactions between gas molecules and sensor materials into electrical signals (such as IV curves), enabling distinctive responses for different gases and facilitating monitoring. Additionally, the use of varied materials produces sensors with diverse responses to gases, showcasing the selectivity of gas-sensitive materials.

Two-dimensional (2D) materials demonstrate immense potential as chemical sensors [5,6,7,8,9,10,11,12], in spintronics applications [13,14,15,16,17,18], and in other exotic applications [19] due to their vast surface-to-volume ratio, potent surface activity, and remarkable electrical conductivity. For instance, Muhmood et al. enhanced the photoelectrocatalytic property of a g-C_3_N_4_ (with a narrow bandgap)/GNP/AgBr heterojunction through collaborative efforts [20]. Fe-ZrO_2_ embedded in g-C_3_N_4_ is reported to exhibit highly efficient photocatalytic degradation of the anti-diabetic drug acarbose (ACB) under visible light [21]. Tao et al. applied 2D black phosphorus as a delivery platform in the field of cancer theranostics [22]. It should be noted that 2D material sensors predominantly operate through the charge-transfer mechanism, with the sensor property hinging on resistivity changes resulting from gas-molecule adsorption. As such, 2D materials are now among the top choices for making toxic gas sensors.

Many experiments have proven that 2D materials such as graphene [14,23,24], MX_2_ [25], and particularly phosphorene and/or metal-doped phosphorene [17,18,26,27,28,29] are excellent sensor materials due to their unique electronic and transport properties [30,31,32,33,34,35,36,37,38,39,40,41,42]. For instance, recent research by Alfalasi et al. analysed 2D-TMD monolayers systematically using DFT calculations and proved that they can be used to create high-performance NO_2_ sensors [43]. However, there are still factors limiting the development of 2D material-based toxic gas sensors, one of which is humidity. At high humidity, the sensitivity of the gas sensor significantly decreases [44,45,46,47]. There are essentially two ways to solve this problem: one is to use desiccants to remove water molecules, and the other is to choose a highly selective material that is not sensitive to water. Clearly, the latter can solve this problem at its root. Therefore, high sensitivity, selectivity, and reusability should be the goals in designing gas sensors. For example, the charge transfer of NO_2_, NO, and H_2_O to (or from) MoS_2_ is 0.10 e, 0.02 e, and 0.01 e [48]; to α-P is 0.20 e, 0.07 e, and 0.04 e [49]; and to graphene is 0.10 e, 0.02 e, and 0.03 e [50], respectively. As can be seen, the amount of charge transfer for NO_2_ is an order of magnitude higher than for NO and H_2_O for all of these materials, indicating that these are good sensor candidates for NO_2_. However, these 2D materials cannot distinguish more toxic NO gases from H_2_O because of their comparable amount of charge transfer. In other words, gas sensors based on these most common 2D materials will be dysfunctional in a humid environment. Furthermore, Kou et al. showed that a large threshold voltage (to overcome the band gap) for semiconducting phosphorene can cause a significant electric field that may lead to a critical Zener breakdown [51]. Thus, it is desirable to find a 2D material that is conducting, sensitive to both NO and NO_2_, and inert to H_2_O. As NO and NO_2_ are paramagnetic but H_2_O is nonmagnetic, it is essential to screen magnetic conducting 2D materials to identify if they can meet the demands required of the desired sensor materials.

**Figure A1:**
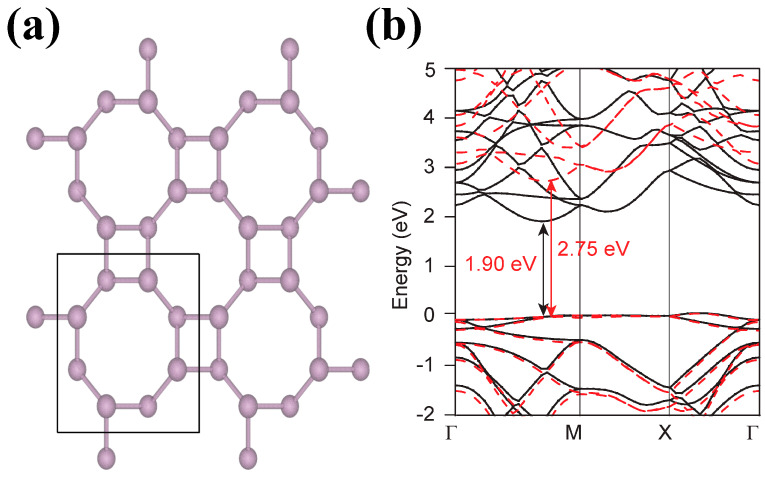
(**a**) The atomic structure of ϵ-phosphorene. The rectangular box represents the unit cell. (**b**) The DFT band structure of ϵ-phosphorene. The PBE (black) gap is 1.90 eV, while the HSE (red) gap is 2.75 eV. The overall shapes of both PEB and HSE are identical.

Phosphorene, also known as black phosphorene (α-phosphorene), has garnered significant interest for a range of applications since its first successful fabrication [15,27,28,52,53,54,55]. Soon after its discovery, researchers proposed various other layered phosphorus allotropes, including blue phosphorus (β-phosphorene) [18,54], γ-phosphorene, and δ-phosphorene [56,57]. In 2021, the feasibility of using green phosphorus as a gas-sensitive material was demonstrated by Kaewmaraya et al., with their sensor results showing high sensitivity and selectivity for NO_2_ [35]. Recently, a new square–octagon phosphorus allotrope has emerged, comprising alternating square and octagon structures, referred to as ϵ-phosphorene, as depicted in Figure A1a [58,59,60,61]. Previous works have reported that both pristine and metal-doped monolayer ϵ-phosphorenes are dynamically, thermodynamically, and mechanically stable [58,59,60,61,62]. The pristine monolayer ϵ-phosphorene is a non-magnetic semiconductor, as shown in Figure A1b. However, Wang et al. found that V-doped octahedral–tetragonal-phase phosphorene (V-ϵ-phosphorene) has a stable ferromagnetic ground state with high Curie temperature [62].

In this work, we examine the adsorption of NO_2_, NO, and H_2_O molecules on a V-ϵ-phosphorene nanosheet using first principles and investigate their sensor performance through IV curve calculations. We first calculate the adsorption energies of these gas molecules to identify their optimal adsorption position and orientation. Subsequently, we undertake a thorough analysis of charge transfer between the molecules and the V-ϵ-phosphorene nanosheet, enabling us to discern the donor or acceptor nature of the molecular dopant. Lastly, to explore the possibilities of using the V-ϵ-phosphorene nanosheet as sensors, we perform a study on the transport properties with the introduction of gas molecules using the non-equilibrium Green’s function method. By observing the differences in IV characteristics among a series of V-ϵ-phosphorene systems, we can gain a basic understanding of the sensitivity and selectivity of this sensor.

## 2. Computational Details

All the calculations were carried out via first-principles methods based on density functional theory (DFT) [63,64], as implemented in the *Vienna Ab Initio Simulation Package* (VASP) [65,66]. The exchange correlation energy was simulated using generalised gradient approximation (GGA) in the form of the Perdew–Burke–Ernzerhof approximation (PBE) [67] functional, while the projector augmented wave (PAW) [68] approximation was used to describe the core electrons as external potentials to the orbitals of study.

In our calculations of the adsorption energy and charge transfer of gas molecules on the V-ϵ-phosphorene substrate, the k-points mesh used for calculation carried out in a 2×2 supercell was 3×3×1. Spin-polarised calculations were performed throughout the work. The kinetic energy cutoff for the plane wave basis set was chosen to be 255 eV, which yielded well-converged total energies. All the structures were relaxed until the remaining force on each atom was reduced to less than 0.01 eV/Å. After calculating the project density of states (PDOS) of the host matrix with the adsorbed NO_2_, NO, and H_2_O molecules, the transport properties of two-terminal devices were studied using the atk package to investigate the IV responses to different gas molecules [29,69,70]. The IV characteristics were obtained by the Landauer–Büttiker formula, which can be written as:

$$I\left(V_{b}\right)=G_{0}\int_{\mu_{L}}^{\mu_{B}}T(E,\;V_{b})dE$$

where $G_{0}$ is the unit of quantum conductance, $T(E,V_{b})$ is the transmission probability of an electron incident at an energy *E* under a potential bias $V_{b}$, and the electropotential difference between the two electrodes is $eV_{b}=\mu_{L}-\mu_{R}$.

## 3. Charge Transfer and Adsorption Energy

Figure A1 displays the atomic structure and band structure of ϵ-phosphorene. Each individual cell contains eight phosphorus atoms. These atoms are arranged in a planar mesh structure, formed by combining edge-sharing octet and quaternary rings, as depicted in Figure A1a. Considering that the PBE method tends to underestimate the band gap of the material, we also employed the HSE method to refine the electronic structure. Our calculations, presented in Figure A1b, reveal that while the HSE does notably correct the band gap, it does not significantly change the shapes of the valence and conduction bands.

**Figure 1:**
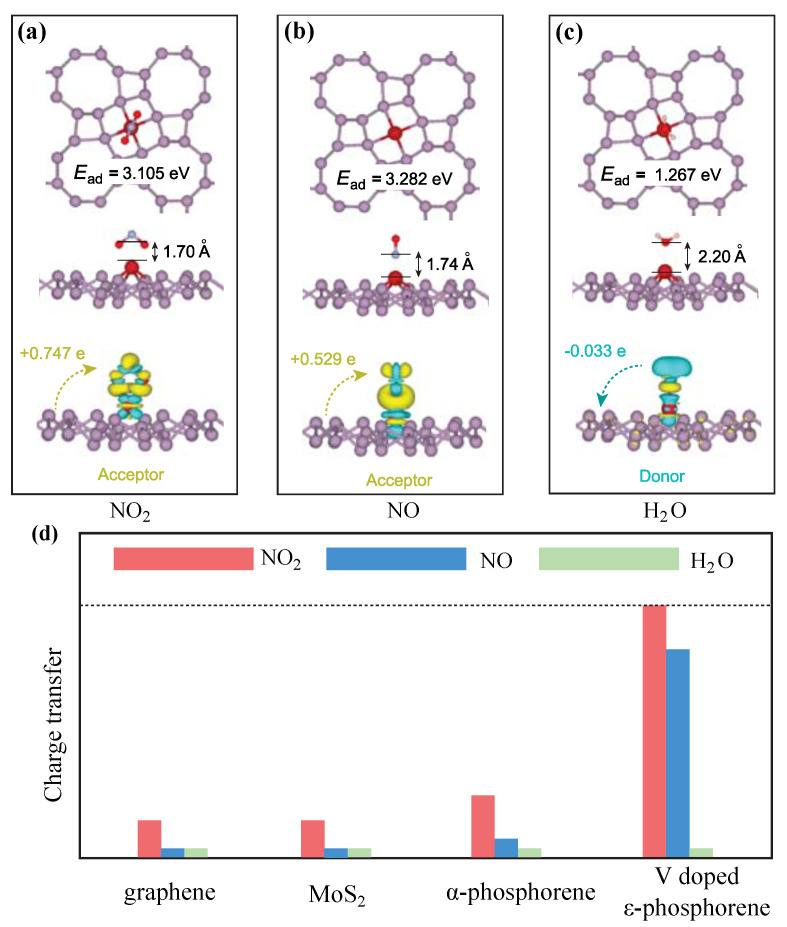
The most favorable adsorption position, distance, and amount of charge transfer of (**a**) NO_2_, (**b**) NO, and (**c**) H_2_O. NO_2_ and NO are electron acceptors, while H_2_O is an electron donor. (**d**) The comparison of charge transfer of graphene, MoS_2_, α-phosphorene, and V-ϵ-phosphorene. Based on the amount of charge transfer, V-doped ϵ-phosphorene can identify both NO_2_ and NO from H_2_O, while most common 2D materials cannot differentiate NO and H_2_O.

Next, we studied the adsorption of two toxic paramagnetic greenhouse gases (NO_2_, NO) and one non-magnetic gas (H_2_O) on top of the V-ϵ-phosphorene. We started with the simplest two-atom NO molecule. The possible orientations of the NO molecule with respect to the ϵ-phosphorene surface were examined; these were, starting from the N atom, the N-O bonds pointing up (u), down (d), or parallel (p) to the ϵ-phosphorene substrate surface. After optimisation, we found that the pointing-up (u) orientation is the most stable, with the highest adsorption energy. The parallel (p) orientation of NO molecules adjusts itself during geometry optimisation and converges to the (u) orientation, which is of lower energy. On the other hand, NO that started with the pointing-down (d) orientation will not adjust itself to the (u) orientation. Nevertheless, the adsorption energy associated with this state is lower than its (u) orientation counterpart, suggesting it is a less stable orientation. Using a Bader analysis, we found that a charge transfer from the V-ϵ-phosphorene monolayer to the NO molecule occurs when the gas molecule is adsorbed. The NO molecule acts as an electron acceptor in this case. When NO is placed in its most stable adsorption orientation, i.e., the upwards (u) orientation, the amount of charge transferred is ΔQ=0.529 e. The charge transfer can be observed more clearly in Figure 1, in which a yellow colour indicates an increase in electrons and a blue colour indicates the depletion of electrons in the region.

The orientations of the NO_2_ molecule with respect to the ϵ-phosphorene surface wee examined; starting from the N atom, we assessed the N-O bonds pointing up (u), down (d), or parallel (p) to the substrate surface. After optimisation, we found that the pointing-down (d) orientation is the most stable. The parallel (p) orientation of the NO_2_ molecules adjusts itself during geometry optimisation, converging to the down (d) orientation, which is of lower energy. On the other hand, NO_2_ that started with a pointing-up (u) orientation will not adjust itself to the (d) orientation. Nevertheless, the adsorption energy associated with this state is lower than that of its (d) orientation counterpart, suggesting it is a less stable orientation. Using a Bader analysis, we found that a charge transfer from the V${}_{\epsilon }$-phosphorene monolayer to the NO_2_ molecule occurs when the gas molecule is adsorbed. The NO_2_ molecule acts as an electron acceptor in this case. When NO_2_ is placed in its most stable adsorption orientation, i.e., the downwards (d) orientation, the amount of charge transferred is ΔQ=0.747 e. The charge transfer can be observed more clearly in Figure 1, in which a yellow colour indicates an increase in electrons and a blue colour indicates the depletion of electrons in the region.

The orientations of the H_2_O molecule with respect to the ϵ-phosphorene surface are examined; starting from the O atom, we examined the O-H bonds pointing up (u), down (d), or parallel (p) to the substrate surface. After optimisation, we found that the most stable orientation is one that lies between the up (u) type and the parallel (p) type. The three atoms in an H_2_O molecule in this orientation lie in a plane that is slightly tilted from the normal plane of the ϵ-phosphorene surface (referred to as the tilted (t) orientation here). The down (d) and parallel (p) orientation of H_2_O molecules adjust themselves during geometry optimisation and converge to the tilted (t) orientation, which is of lower energy. On the other hand, H_2_O that started with a pointing-up (u) orientation will not adjust itself to the (t) orientation. Nevertheless, the adsorption energy associated with this state is slightly lower than its (t) orientation counterpart, suggesting it is a less stable orientation. Using a Bader analysis, we found that a charge transfer from the molecule to the V-ϵ-phosphorene monolayer occurs when the H_2_O gas molecule is adsorbed. The H_2_O molecule acts as an electron donor in this case. When H_2_O is placed in its most stable adsorption orientation, i.e., the tilted orientation between parallel and straight up, the amount of charge transferred is ΔQ=−0.033 e. The charge transfer can be observed more clearly in Figure 1, in which a yellow colour indicates an increase in electrons and a blue colour indicates the depletion of electrons in the region.

The charge transfer analysis shows that the amount of charge transferred (ΔQ) is 0.529 e, 0.747 e, and −0.033 e for the NO, NO_2_, and H_2_O molecules, respectively (Figure 1). As can be seen, there is more charge transfer between the substrate and NO/NO_2_ molecules than with H_2_O. This is because, compared to the adsorption of non-magnetic H_2_O molecules, the magnetic NO_2_ and NO molecules are able to bond more strongly to the V atom due to the magnetic coupling effect. Moreover, both magnetic gas molecules act as electron acceptors when adsorbed onto the V-ϵ-phosphorene monolayer.

**Figure 2:**
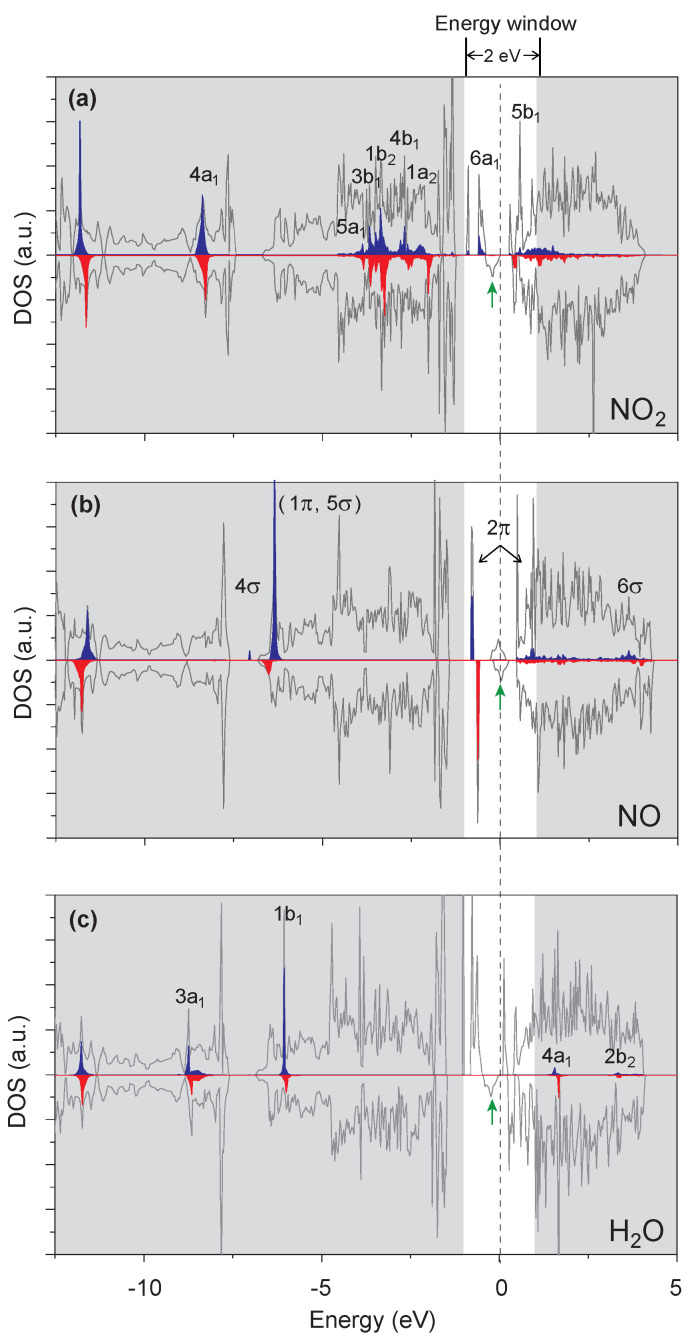
Projected density of states of V${}_{\epsilon }$-phosphorene after adsorption of (**a**) NO_2_, (**b**) NO, and (**c**) H_2_O. Within the energy window of 2 eV near the Fermi level, there is a strong hybridisation of the localised spin states of the host with the molecular frontier orbitals of NO_2_ and NO, while the molecular orbitals of H_2_O are located far away from the Fermi level. The electronic structure near the Fermi level is modified by the adsorption of NO_2_ and NO. The green arrows indicate the sin states of vanadium. The blue and red peaks are the molecular frontier orbitals.

In order to understand the adsorption properties of these molecules on ϵ-phosphorene surfaces, we calculated the project density of the states of these adsorption systems and the molecular frontier orbitals of the adsorbed molecules (Figure 2). It is well-known that the highest occupied molecular orbital (HOMO) and the lowest unoccupied molecular orbital (LUMO) play a crucial role during interactions with the surface of substrates in the vicinity of the Fermi level. Using NO as an example (Figure 2b), after adsorption on the V${}_{\epsilon }$-phosphorene monolayer, the LUMO peaks of the NO molecule are shifted below the Fermi level (2π peaks within the energy window) through mixing with the V atom orbitals (the green arrow within the energy window) that are of a similar energy. Electrons that previously stay in the orbitals of V atoms travel to the LUMO of NO molecules, lowering the system energy and stabilising the adsorption of NO molecules. Overall, there is a strong hybridisation of the localised spin state of the substrate with the molecular frontier orbitals of NO near the Fermi level (Figure 2b). This analysis is also in agreement with the adsorption energy calculations, adsorption distance, and charge transfer. Figure 2a shows that NO_2_ has a similar hybridisation behaviour. However, the H_2_O molecule is free of hybridisation in either HOMO or LUMO with the state of the substrate (the green arrow in Figure 2c), which again proves that the adsorption of NO and NO_2_ by the material is stronger than that of H_2_O.

## 4. IV Responses of Sensors

The amount of charge transferred between the adsorbed gas molecule and the V-ϵ-phosphorene substrate is an indicator that reflects the influence of gas molecule adsorption on the transport properties of a V-ϵ-phosphorene monolayer. However, the exact amount of charge transferred is not the interest of experiments and sensor designs because the relative amount of charge transferred can better describe the capability of electron transport. A more direct parameter to look at is the current–voltage (IV) character with and without the adsorption of gas molecules. As the adsorption-induced charge transfer will alter the resistivity of the system, the current flow through the monolayer is supposed to be different for different adsorbates under the same applied voltage.

**Figure A2:**
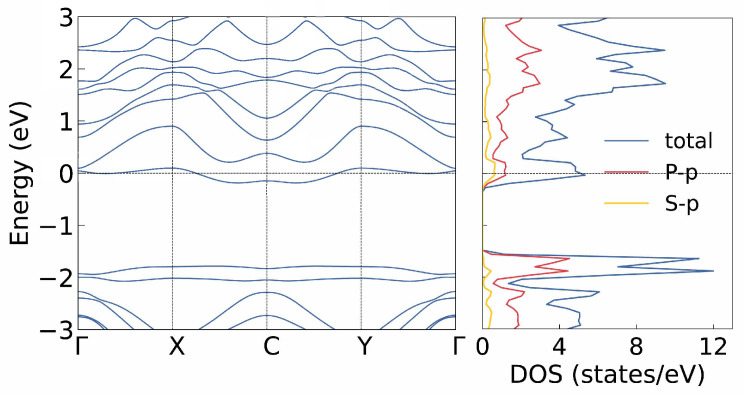
The DFT band structure and DOS of S-ϵ-phosphorene. The energy band near 0 eV is partly occupied as the Fermi level crosses this band, which allows for the supply of conductive electrons.

To study the IV relationship of gas-molecule-adsorbed V-ϵ-phosphorene monolayers, a two-probe system was developed. In this system, the left and right semi-infinite electrode regions were in contact with a 2×2 V-ϵ-phosphorene supercell that served as the central scattering region. In this work, a *metallic* sulphur-doped ϵ-phosphorene monolayer was used as the electrodes. As shown in Figure A2, the band structure of the S-ϵ-phosphorene monolayer demonstrates that S-ϵ-phosphorene behaves as a conductor. When a bias voltage is applied, the Fermi level of the left electrode moves upwards with respect to the right electrode, injecting electrons into the system. The use of S-ϵ-phosphorene as an electrode ensures near-perfect interfacial contact, as the lattice size of the S-ϵ-phosphorene unit cell is essentially the same as that of the ϵ-phosphorene unit cell. This setup also greatly simplifies calculations for geometry optimisation, as a perfect contact will not induce significant distortion at the interface.

**Figure 3:**
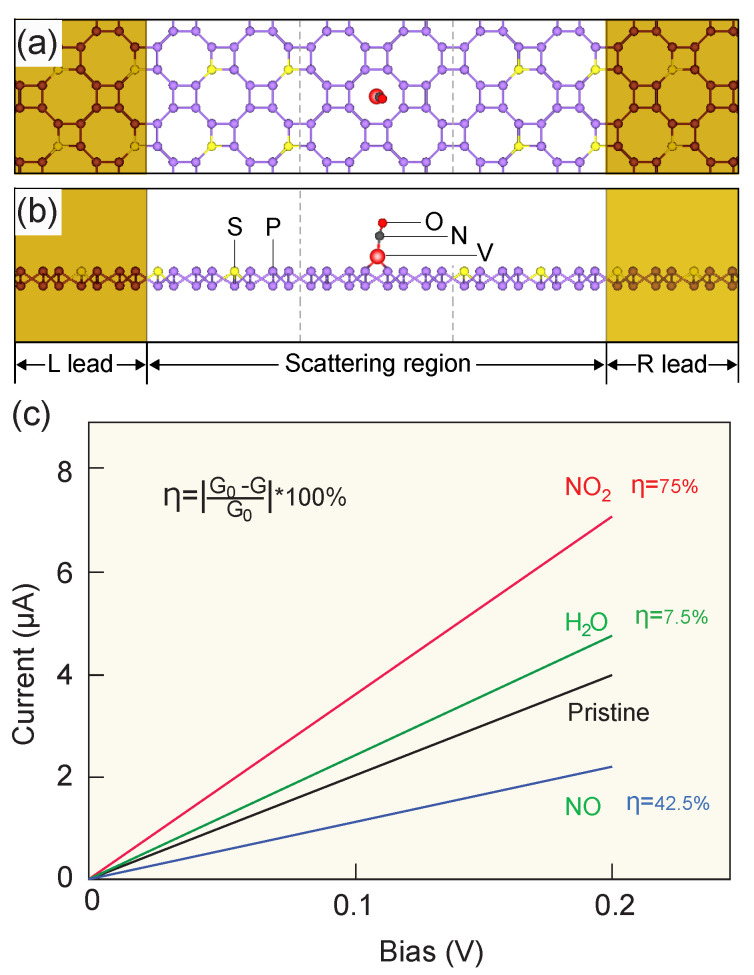
(**a**) Top and (**b**) side view of a two-terminal device with adsorption of a NO molecule. Metallic S-decorated ϵ-phosphorene is used as the electrode. (**c**) The I–V curve of pristine, NO_2_-, NO-, and H_2_O-adsorbed V-ϵ-phosphorene. The change in gradient (conductance *G*) of NO_2_, NO, and H_2_O is 75%, 42.5%, and 7.5%, respectively.

**Figure A3:**
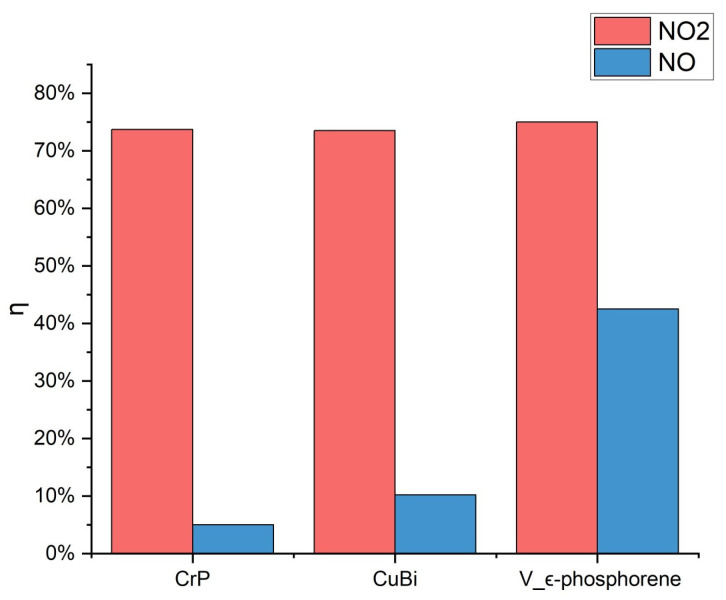
The comparison of relative change of conductance of NO and NO${}_{2}$ between CrP [72], CuBi [71], and V-ϵ-phosphorene used in this work.

From the IV relationship depicted in Figure 3, it can be observed that when a bias voltage is applied, there is an immediate current flow through the device because of the metallic spacer. The IV relationship of the V-ϵ-phosphorene monolayer shows a distinction compared to the IV relations of NO_2_ and NO, similar to that of H_2_O. This is because NO and NO_2_ have a stronger adsorption capacity on the surface of the material than H_2_O. This means that more NO and NO_2_ molecules will adsorb on the surface of the material, forming more carriers and thus increasing the relative conductivity. However, when NO molecules are adsorbed, an interaction between N atoms and V atoms occurs (Figure 1), and N atoms take up more electrons. This results in the fact that, after the adsorption of NO, the free electrons originally used for conducting electricity are partially transferred to the adsorbed NO molecule, reducing the number of carriers available for conducting a current. This explains why the conductivity decreases instead after NO adsorption. The calculated relative change in the conductance of NO_2_, NO, and H_2_O is 74%, 42.5%, and 7.5%, respectively. Upon comparing these performance values with other materials, including CuBi [71] and CrP [72], we found that in the case of similar relative changes in the conductance of NO_2_, the sensing performance of our material for NO is better than the other two materials (Figure A3). Furthermore, our V-ϵ-phosphorene monolayer can achieve its level of performance with a notably small voltage, which means it can reduce energy consumption and the risk of the electrochemical or thermal failure of materials. This shows that V-ϵ-phosphorene monolayers are a good candidate for the sensing of toxic NO_2_ and NO even in humid environments.

## 5. Electric Field Effect

**Figure 4:**
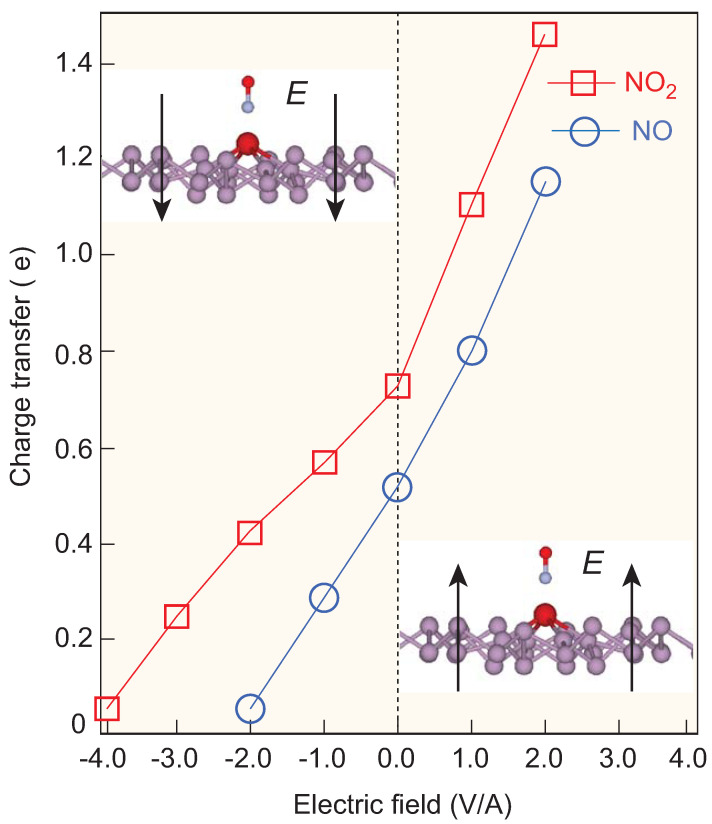
The effect of the electric field on the charge transfer of NO_2_ and NO. The upward (downward) direction of the electric field is positive (negative). The positive electric field can enhance the charge transfer and thus the sensitivity of the device, while the negative electric field can reduce the charge transfer and thus remove the adsorbed molecules for repeated use.

The charge transfer between molecules and the 2D host matrix is the driving mechanism of gas sensors. A greater charge transfer results in greater changes in the electronic structure and resistance of the host. In other words, sensors with more charge transfer capabilities are more sensitive. However, a great amount of charge transfer results in the strong adsorption of molecules on the sheet. A strong bonding of gas molecules onto the sensor suggests that the adsorption process is irreversible, thus making the sensor not suitable for repeated use. If a given material is to be made into a sensor, it is important for it to have an appropriate adsorption energy to allow both the adsorption and desorption processes to happen, or alternatively, the adsorption strength of the molecules on the sensor must be tunable. For example, there may be more charge transfer during the detection process and less charge transfer during the cleaning process. As we know, most gas molecules have an intrinsic dipole moment, which can be tuned by an external dipole effect, such as electric fields [16,73]. Figure 4 shows that different polar directions of an external electric field can change the amount of charge transferred between the molecule and sensor. Thus, applying a gate voltage is a realistic way to tune the performance of gas sensors.

## 6. Limitations and Future Scope

Before concluding, we would like to discuss some limitations and possible future directions for ϵ-phosphorene-based gas sensors. The computational calculations for this system are costly due to the large size of the device configurations. Extensive efforts have been made to explore various parameters, and a significant amount of time has been invested in this process. Furthermore, the synthesis of the material and the construction of the device may encounter several issues, including material stability, the feasibility of device construction, and the impact of environmental factors. In particular, there may be defects in the process of material preparation, leading to experimental sensing results that are not as expected. In addition, the presence of multiple gases in the atmosphere and their interaction with the material may affect the results of the experiments. Looking forward, future research may aim to investigate the sensor capabilities of V-ϵ-phosphorene monolayers with respect to other gas molecules, such as SO_2_, SO, and others. We also encourage the experimental synthesis of ϵ-phosphorene and its transformation into a tangible product for further testing and evaluation.

## 7. Conclusions

In conclusion, exposure to high humidity can render a gas sensor dysfunctional as the sensor may also be sensitive to H_2_O molecules and thus may not be able to distinguish changes in IV responses resulting from NO/NO_2_ or from H_2_O. Furthermore, the presence of a large threshold voltage in semiconducting nanodevices can generate a significant electric field and potentially lead to critical Zener breakdown. Consequently, this study aims to guide the design of 2D gas sensors using a conductive 2D material that is sensitive to specific molecules while being inert to ambient gases.

Our first-principle calculations indicate that both NO and NO_2_, being paramagnetic, exhibit high adsorption energy for the magnetic V-ϵ-phosphorene monolayer. This value is hundreds of times larger than that of H_2_O, as determined by the amount of charge transfer that occurs. Our transport calculations demonstrate a distinguished IV response to NO/NO_2_ compared with H_2_O. These results suggest that V-ϵ-phosphorene monolayers are an ideal candidate for sensor designs intended for the detection of toxic NO and NO_2_ gases, even in a humid environment. Additionally, we have found that the gate voltage in three-terminal devices can effectively modulate the charge transfer. The more charge transferred under a positive field, the more the resistance changes, ensuring that the sensor remains sensitive under conditions of low gas concentrations. Conversely, the less charge transferred, the lower the molecule’s adsorption energy; this then allows the adsorbed molecules to be removed from the sensor’s surface, facilitating its repeated use. 

## Data Availability

Not applicable.

## Sample Availability

Samples of the compounds are available from the authors.
